# The *Amblyomma maculatum* Koch, 1844 (Acari: Ixodidae) group of ticks: phenotypic plasticity or incipient speciation?

**DOI:** 10.1186/s13071-018-3186-9

**Published:** 2018-11-29

**Authors:** Paula Lado, Santiago Nava, Leonardo Mendoza-Uribe, Abraham G. Caceres, Jesus Delgado-de la Mora, Jesus D. Licona-Enriquez, David Delgado-de la Mora, Marcelo B. Labruna, Lance A. Durden, Michelle E. J. Allerdice, Christopher D. Paddock, Matias P. J. Szabó, José M. Venzal, Alberto A. Guglielmone, Lorenza Beati

**Affiliations:** 1 U. S. National Tick Collection, Institute for Coastal Plain Science, Georgia Southern University, Statesboro, GA 30460 USA; 2 Present address: Department of Evolution, Ecology, and Organismal Biology, The Ohio State University, Columbus, OH 43212 USA; 3 Instituto Nacional de Tecnologia Agropecuaria, Estación Experimental Agropecuaria Rafaela, Santa Fe, Argentina; 4 Laboratorio de Entomología, Instituto Nacional de Salud, Lima, Peru; 5 Departamento Académico de Microbiologia Médica, Facultad de Medicina, Universidad Nacional Mayor de San Marcos, Lima, Peru; 6 Laboratorio de Entomología, Instituto Nacional de Salud, Lima, Peru; 7 Department of Medicine and Health Sciences, University of Sonora, Sonora, Mexico; 8 Department of Agronomic and Veterinary Sciences, Technologic Institute of Sonora, Ciudad Obregón, Sonora Mexico; 9 Departamento de Medicina Veterinária Preventiva e Saúde Animal, Faculdade de Medicina Veterinária e Zootecnia, Universidade de São Paulo, São Paulo, Brazil; 10 Department of Biology, Georgia Southern University, Statesboro, GA 30460 USA; 11 Rickettsial Zoonoses Branch, National Center for Emerging and Zoonotic Infectious Diseases, Centers for Disease Control and Prevention, Atlanta, GA USA; 12 Laboratório de Ixodologia, Faculdade de Medicina Veterinária, Universidade Federal de Uberlândia, Uberlândia, Minas Gerais Brazil; 13 Departamento de Parasitología Veterinaria, Facultad de Veterinaria, Universidad de la República, Regional Norte - Salto, Rivera 1350, 50000 Salto, CP Uruguay

**Keywords:** *Amblyomma*, Systematics, Ticks, *Amblyomma maculatum*, Taxonomic reassessment, Phylogenetic analysis, *Amblyomma triste*

## Abstract

**Background:**

The goal of this study was to reassess the taxonomic status of *A. maculatum*, *A. triste* and *A. tigrinum* by phylogenetic analysis of five molecular markers [four mitochondrial: *12S* rDNA, *16S* rDNA, the control region (DL) and cytochrome *c* oxidase 1 (*cox*1), and one nuclear: ribosomal intergenic transcribed spacer 2 (ITS2)]. In addition, the phenotypic diversity of adult ticks identified as *A. maculatum* and *A. triste* from geographically distinct populations was thoroughly re-examined.

**Results:**

Microscopic examination identified four putative morphotypes distinguishable by disjunct geographical ranges, but very scant fixed characters. Analysis of the separated mitochondrial datasets mostly resulted in conflicting tree topologies. Nuclear gene sequences were almost identical throughout the geographical ranges of the two species, suggesting a very recent, almost explosive radiation of the terminal operational taxonomic units. Analysis of concatenated molecular datasets was more informative and indicated that, although genetically very close to the *A. maculatum - A. triste* lineage, *A. tigrinum* was a monophyletic separate entity. Within the *A. maculatum - A. triste* cluster, three main clades were supported. The two morphotypes, corresponding to the western North American and eastern North American populations, consistently grouped in a single monophyletic clade with many shared mitochondrial sequences among ticks of the two areas. Ticks from the two remaining morphotypes, south-eastern South America and Peruvian, corresponded to two distinct clades.

**Conclusions:**

Given the paucity of morphological characters, the minimal genetic distance separating morphotypes, and more importantly the fact that two morphotypes are genetically indistinguishable, our data suggest that *A. maculatum* and *A. triste* should be synonymized and that morphological differences merely reflect very recent local adaptation to distinct environments in taxa that might be undergoing the first steps of speciation but have yet to complete lineage sorting. Nonetheless, future investigations using more sensitive nuclear markers and/or crossbreeding experiments might reveal the occurrence of very rapid speciation events in this group of taxa. Tentative node dating revealed that the *A. tigrinum* and *A. maculatum - A. triste* clades split about 2 Mya, while the *A. maculatum - A.triste* cluster radiated no earlier than 700,000 years ago.

**Electronic supplementary material:**

The online version of this article (10.1186/s13071-018-3186-9) contains supplementary material, which is available to authorized users.

## Background

The *Amblyomma maculatum* group includes, according to Camicas et al. [1], the following species: *A. maculatum* Koch, 1844; *Amblyomma neumanni* Ribaga, 1902; *Amblyomma parvitarsum* Neumann, 1901; *Amblyomma tigrinum* Koch, 1844 and *Amblyomma triste* Koch, 1844. Together with the *Amblyomma ovale* Koch, 1844 group which encompasses *A. ovale* and *Amblyomma aureolatum* (Pallas, 1772), they have been clustered by Camicas et al. [1] in a revised version of subgenus *Anastosiella*, originally erected by Santos Dias [2], who also included within this subgenus *Amblyomma pecarium* Dunn, 1933 and *Amblyomma brasiliense* Aragão, 1908. However, within the *A. maculatum* group, the adult and immature stages of *A. neumanni* and *A. parvitarsum* are morphologically distinguishable from the other species. Unlike the other three taxa, they are both characterized by incomplete marginal grooves in males, with *A. parvitarsum* having beady and orbited eyes [3]. In females, aside from *A. neumanni*, all species are glabrous dorsally. *Amblyomma parvitarsum* females also have beady and orbited eyes. Other diagnostic differences are listed in Estrada- Peña et al. [3], who suggested that *A. neumanni* and *A. parvitarsum* should be grouped with the *A. ovale* group in a yet to be determined subgenus, with *A. maculatum*, *A. triste* and *A. tigrinum* the only remaining members of the subgenus *Anastosiella.*

Members of the subgenus *Anastosiella* are morphologically very similar. This similarity is more apparent between *A. maculatum* and *A. triste*, and is examined in depth in this study. Koch [4] briefly described the three taxa based on males of *A. maculatum* and *A. tigrinum*, and a female of *A. triste.* He completed his description in 1850 [5] and essentially reported differences in punctation and ornamentation. Neumann [6] synonymyzed *A. tigrinum* and *A. triste* with *A. maculatum* after failing to observe differences in the shape of distal spines (modified setae) on tibiae II to IV (called tarsi by [6], protarsi by [7] and metatarsi by [8]). Kohls [8] reestablished *A. tigrinum* and *A. triste* as valid species and completely redescribed the three taxa. In that work, the characters differentiating the three species were the presence/absence of tubercles on the festoons, and the presence of one or two spurs on “metatarsi” of legs II-IV. Nevertheless, the identification chiefly of *A. maculatum* and *A. triste* has consistently been challenging [3, 9–11] and marred by frequent misidentifications [12–14]. Taxonomic issues are not limited to adult stages: immatures, for which taxonomic keys are available, are even more difficult to differentiate [3, 11, 15–17] with, again, the exception of *A. parvitarsum* and *A. neumanni* which are easily separated from the other three species*.*

The distribution of *A. maculatum* is presumably confined to the southern USA, Central America and some areas of Colombia, Venezuela, Peru and Ecuador, whereas *A. tigrinum* is reported to occur only in South American countries [8, 18–21]. *Amblyomma triste* was regarded as being exclusively South American in its distribution until recently, when it was reported from Mexico and the southwestern USA [11, 22], thus increasing the number of tick species with both, a Neotropical and Nearctic distribution [9].

A thorough review of the taxonomic status of this complex of species is essential, not only for systematic reasons, but also because *A. maculatum*, *A. triste* and *A. tigrinum* are recognized increasingly as vectors of pathogens of veterinary and public health importance throughout the Americas, particularly *Rickettsia parkeri* [23–34] and *Hepatozoon americanum* [35].

Molecular techniques used to infer phylogenetic relationships and evaluate the taxonomic status of the different species of the *A. maculatum* group have yet to be applied in a comprehensive manner. Preliminary reports based on the analysis of *16S* rDNA sequences confirmed that *A. maculatum*, *A. triste* and *A. tigrinum* are closely related to each other, whereas *A. neumanni* and *A. parvitarsum* are distinct not only from each other and from the rest of the *A. maculatum* group of taxa, but do not cluster with the *A. ovale* group of species [3].

The aim of this study was to reassess the taxonomic status of *A. maculatum*, *A. triste* and *A. tigrinum* by analyzing their phylogenetic relationships determined by comparisons of one nuclear and four mitochondrial gene sequences. In addition, a comprehensive morphological analysis of the adult stage of *A. maculatum* and *A. triste* is presented. Our working hypothesis is that *A. maculatum*, *A. triste* and *A. tigrinum* comprise three separate species.

## Results

### Morphological reassessment of the *A. maculatum - A. triste* specimens

**Fig. 1:**
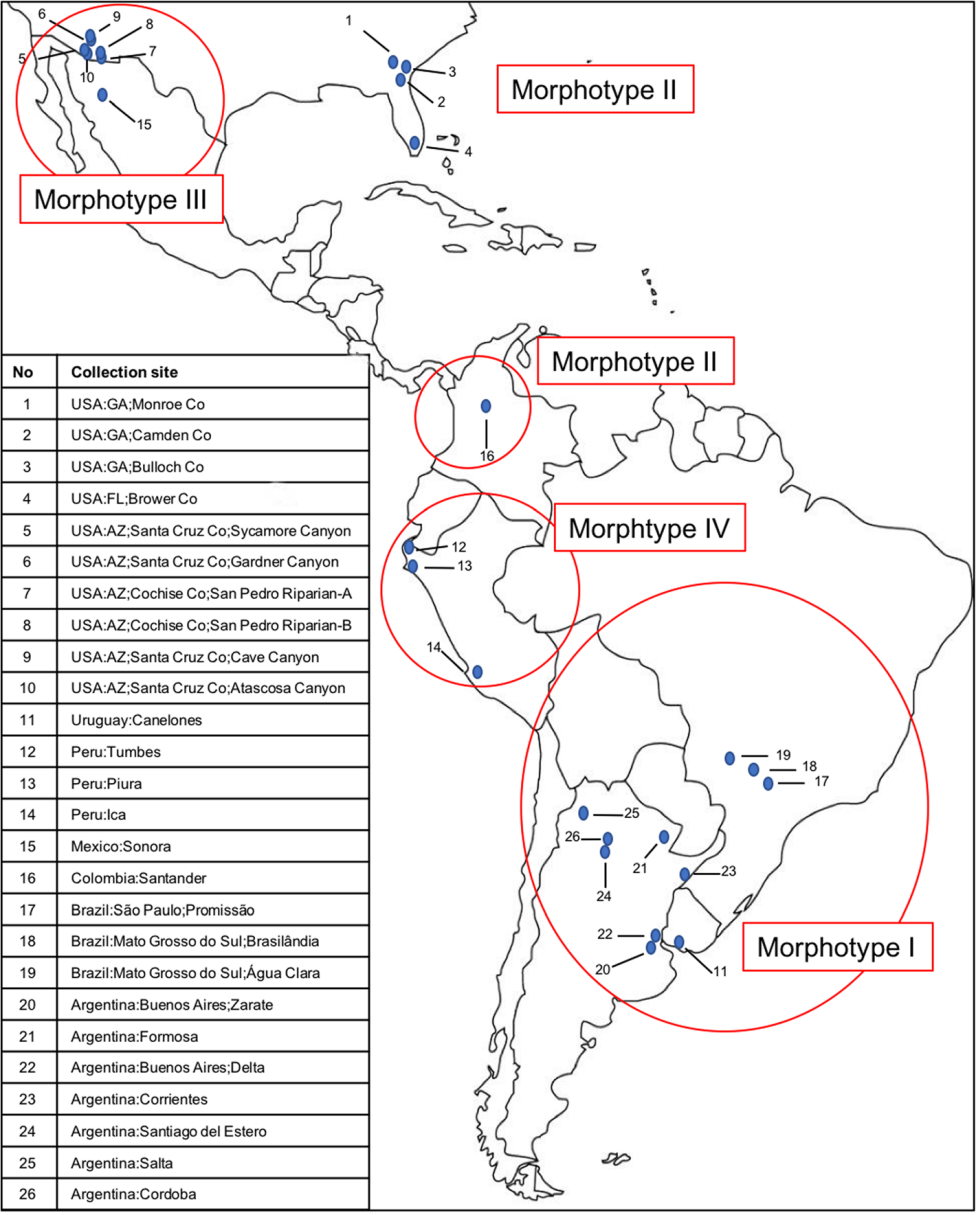
Map showing the geographical locations of ticks used for molecular analysis in this study and their corresponding morphotypes

Microscopic examination identified four morphological groups that, for the sake of simplicity, will be called here Morphotypes I, II, III and IV. The four morphotypes have disjunct geographical distributions (Fig. 1; morphotype distribution for the samples used in the molecular analyses) and feature many common and a few distinctive character combinations (Table 1). The shape of modified setae on the ventral distal end of the tibiae of legs II-IV (tibial armature) have been commonly used to differentiate ticks of the *A. maculatum* group. They have been called spines, setae or spurs in earlier literature. In the following descriptions we will use the term “seta” if the structure is fine and weakly sclerotized, “spine” if the structure is thick and heavily sclerotized. For measurements, refer to scales on the images.

**Table 1:** Tick samples used for the molecular portion of this study and the gene amplifications results. Crosses indicate successful amplification and sequencing of the corresponding molecular marker

Species	Mt	Voucher ID	Country	State/ province/ department	Locality	Code	Coordinates	*12S*	*16S*	DL	ITS2	*cox*1	*cox*2
*A. neumanni*	na		Argentina	Cordoba	Dean Funes	AN	30°26'S, 64°20'W	×	×	×	×	×	×
*A. parvitarsum*	na		Argentina	Salta	45 km E of S. Antonio de los Cobres	AP	24°22'S, 66°42'W	×	×		×	×	×
*A. tigrinum*	na		Argentina	SDE	Pozo Hondo	SDE_16A	27°10'S, 64°30'W	×	×		×	×	×
*A. tigrinum*	na		Argentina	SDE	Pozo Hondo	SDE_16B	27°10'S, 64°30'W	×	×	×	×	×	
*A. triste*	I		Argentina	Corrientes	Colonia Pellegrini	CR_15A	29°00'S, 57°30'W	×	×	×	×	×	
*A. triste*	I	INTA 1979	Argentina	BA	Inta Delta	BA_AT3A	34°11'S, 58°50'W	×				×	
*A. triste*	I	INTA 1980	Argentina	BA	Inta Delta	BA_AT3B	34°11'S, 58°50'W					×	
*A. triste*	I	INTA 1981	Argentina	BA	Inta Delta	BA_AT3C	34°11'S, 58°50'W	×				×	
*A. triste*	I	INTA 1982	Argentina	BA	Inta Delta	BA_AT3D	34°11'S, 58°50'W					×	×
*A. triste*	I	INTA 1983	Argentina	BA	Inta Delta	BA_AT3E	34°11'S, 58°50'W			×		×	
*A. triste*	I	INTA 1984	Argentina	BA	Inta Delta	BA_1A	34°11'S, 58°50'W	×	×	×	×	×	
*A. triste*	I	INTA 1985	Argentina	BA	Inta Delta	BA_1A2	34°11'S, 58°50'W	×	×	×		×	
*A. triste*	I	INTA 1986	Argentina	BA	Inta Delta	BA_1B	34°11'S, 58°50'W	×	×	×	×	×	
*A. triste*	I	INTA 1987	Argentina	BA	Inta Delta	BA_1C	34°11'S, 58°50'W	×	×	×	×	×	
*A. triste*	I	INTA 1988	Argentina	BA	Inta Delta	BA_1D	34°11'S, 58°50'W		×		×	×	
*A. triste*	I	INTA 1989	Argentina	BA	Inta Delta	BA_1E	34°11'S, 58°50'W		×		×	×	
*A. triste*	I	INTA 1990	Argentina	BA	Inta Delta	BA_18A	34°11'S, 58°50'W	×	×	×		×	
*A. triste*	I	INTA 1991	Argentina	BA	Inta Delta	BA_18B	34°11'S, 58°50'W	×	×	×	×		
*A. triste*	I	INTA 1992	Argentina	BA	Inta Delta	BA_18C	34°11'S, 58°50'W	×	×	×	×		
*A. triste*	I	INTA 2096	Argentina	Formosa	Reserva El Bagual	FO_12A	26°10'S, 58°56'W	×	×	×	×	×	×
*A. triste*	I	INTA 2097	Argentina	Formosa	Reserva El Bagual	FO_12B	26°10'S, 58°56'W		×	×		×	
*A. triste*	I	INTA 2098	Argentina	Formosa	Reserva El Bagual	FO_12C	26°10'S, 58°56'W		×	×	×		
*A. triste*	I	INTA 2099	Argentina	Formosa	Reserva El Bagual	FO_12D	26°10'S, 58°56'W	×	×	×	×		
*A. triste*	I	INTA 2100	Argentina	Formosa	Reserva El Bagual	FO_12E	26°10'S, 58°56'W	×	×	×	×		
*A. triste*	I		Argentina	BA	Zarate	BA_AT1A	34°05'S, 59°00'W				×	×	
*A. triste*	I		Argentina	BA	Zarate	BA_AT1B	34°05'S, 59°00'W	×				×	
*A. triste*	I		Argentina	BA	Zarate	BA_AT1C	34°05'S, 59°00'W	×				×	
*A. triste*	I		Argentina	BA	Zarate	BA_17A	34°05'S, 59°00'W	×	×		×	×	
*A. triste*	I		Argentina	BA	Zarate	BA_17B	34°05'S, 59°00'W	×	×	×	×	×	
*A. triste*	I		Argentina	BA	Zarate	BA_17C	34°05'S, 59°00'W	×	×	×	×	×	
*A. tigrinum*	na		Brazil	Goias	Caldas Novas	GO_11A	17°45'S, 48°38'W	×	×	×		×	
*A. tigrinum*	na		Brazil	Goias	Mineiros	GO_5A	17°34'S, 52°33'W	×	×	×		×	×
*A. tigrinum*	na		Brazil	Goias	Mineiros	GO_5A2	17°34'S, 52°33'W	×	×	×		×	
*A. tigrinum*	na		Brazil	Goias	Mineiros	GO_5B	17°34'S, 52°33'W	×	×			×	
*A. tigrinum*	na		Brazil	Goias	Mineiros	GO_5C	17°34'S, 52°33'W	×	×	×		×	×
*A. tigrinum*	na		Brazil	Goias	Mineiros	GO_5D	17°34'S, 52°33'W		×	×			
*A. tigrinum*	na		Brazil	Goias	Mineiros	GO_5E	17°34'S, 52°33'W	×	×	×			
*A. tigrinum*	na		Brazil	Goias	Mineiros	GO_3A1	17°34'S, 52°33'W	×	×	×	×	×	
*A. triste*	I	USNMENT1430164	Brazil	MGS	Agua Clara	MGS_6A	20°14'S, 53°23'W	×	×		×		
*A. triste*	I	USNMENT1430165	Brazil	MGS	Agua Clara	MGS_6A2	20°14'S, 53°23'W	×	×	×	×	×	
*A. triste*	I	USNMENT1430166	Brazil	MGS	Agua Clara	MGS_6B	20°14'S, 53°23'W	×	×	×	×	×	
*A. triste*	I	USNMENT1430167	Brazil	MGS	Agua Clara	MGS_6C	20°14'S, 53°23'W	×	×	×	×	×	
*A. triste*	I	USNMENT1430168	Brazil	MGS	Agua Clara	MGS_6D	20°14'S, 53°23'W	×	×	×	×	×	
*A. triste*	I	USNMENT1430169	Brazil	MGS	Agua Clara	MGS_6E	20°14'S, 53°23'W	×	×	×	×		
*A. triste*	I	USNMENT1430170	Brazil	MGS	Agua Clara	MGS_6F	20°14'S, 53°23'W		×	×		×	
*A. triste*	I	USNMENT1430171	Brazil	MGS	Agua Clara	MGS_6G	20°14'S, 53°23'W		×	×		×	
*A. triste*	I	USNMENT1430172	Brazil	MGS	Agua Clara	MGS_8A	20°14'S, 53°23'W	×	×	×	×	×	
*A. triste*	I	USNMENT1430173	Brazil	MGS	Agua Clara	MGS_8A2	20°14'S, 53°23'W	×	×	×	×	×	
*A. triste*	I	USNMENT1430174	Brazil	MGS	Agua Clara	MGS_8B	20°14'S, 53°23'W	×	×	×	×	×	
*A. triste*	I	USNMENT1430175	Brazil	MGS	Agua Clara	MGS_8C	20°14'S, 53°23'W	×	×	×	×		
*A. triste*	I	USNMENT1430176	Brazil	MGS	Agua Clara	MGS_8D	20°14'S, 53°23'W		×		×	×	
*A. triste*	I	USNMENT1430177	Brazil	MGS	Agua Clara	MGS_8E	20°14'S, 53°23'W		×	×	×		
*A. triste*	I	USNMENT1430178	Brazil	MGS	Agua Clara	MGS_8F	20°14'S, 53°23'W	×	×	×	×	×	
*A. triste*	I	USNMENT1430179	Brazil	MGS	Agua Clara	MGS_8G	20°14'S, 53°23'W	×	×	×		×	
*A. triste*	I	USNMENT1430180	Brazil	MGS	Agua Clara	MGS_8H	20°14'S, 53°23'W	×	×	×		×	
*A. triste*	I	USNMENT1430181	Brazil	MGS	Agua Clara	MGS_8I	20°14'S, 53°23'W	×	×	×	×		
*A. triste*	I	USNMENT1430182	Brazil	MGS	Agua Clara	MGS_8J	20°14'S, 53°23'W	×	×	×	×		
*A. triste*	I	USNMENT1430183	Brazil	MGS	Agua Clara	MGS_8K	20°14'S, 53°23'W	×	×	×	×		
*A. triste*	I	USNMENT1430184	Brazil	MGS	Agua Clara	MGS_8L	20°14'S, 53°23'W	×	×	×			
*A. triste*	I	USNMENT1430185	Brazil	MGS	Agua Clara	MGS_8M	20°14'S, 53°23'W	×	×	×			
*A. triste*	I	USNMENT1430186	Brazil	MGS	Agua Clara	MGS_8N	20°14'S, 53°23'W	×	×	×	×		
*A. triste*	I	USNMENT1430187	Brazil	MGS	Agua Clara	MGS_8O	20°14'S, 53°23'W	×	×	×			
*A. triste*	I	USNMENT1430188	Brazil	MGS	Agua Clara	MGS_8P	20°14'S, 53°23'W	×	×	×	×		
*A. triste*	I	USNMENT1430189	Brazil	MGS	Agua Clara	MGS_8Q	20°14'S, 53°23'W	×	×	×	×		
*A. triste*	I	USNMENT01430159	Brazil	Sao Paulo	Promissão	SP_AT2A	21°32'S, 49°52'W	×				×	
*A. triste*	I	USNMENT01430160	Brazil	Sao Paulo	Promissão	SP_AT2B	21°32'S, 49°52'W	×		×		×	
*A. triste*	I	USNMENT01430161	Brazil	Sao Paulo	Promissão	SP_AT2C	21°32'S, 49°52'W	×		×			
*A. triste*	I	USNMENT01430162	Brazil	Sao Paulo	Promissão	SP_AT2D	21°32'S, 49°52'W	×					
*A. triste*	I	USNMENT01430163	Brazil	Sao Paulo	Promissão	SP_AT2E	21°32'S, 49°52'W					×	
*A. triste*	I	USNMENT01430164	Brazil	Sao Paulo	Promissão	SP_AT2F	21°32'S, 49°52'W	×		×			×
*A. triste*	I	USNMENT01430165	Brazil	Sao Paulo	Promissão	SP_AT2G	21°32'S, 49°52'W	×				×	
*A. triste*	I	USNMENT01430166	Brazil	Sao Paulo	Promissão	SP_2A	21°32'S, 49°52'W		×				
*A. triste*	I	USNMENT01430167	Brazil	Sao Paulo	Promissão	SP_2B	21°32'S, 49°52'W	×	×			×	
*A. triste*	I	USNMENT01430168	Brazil	Sao Paulo	Promissão	SP_2C	21°32'S, 49°52'W	×	×	×			×
*A. triste*	I	USNMENT01430169	Brazil	Sao Paulo	Promissão	SP_13A	21°32'S, 49°52'W	×	×	×		×	
*A. triste*	I	USNMENT01430170	Brazil	Sao Paulo	Promissão	SP_13B	21°32'S, 49°52'W		×	×	×		
*A. triste*	I	USNMENT01430171	Brazil	Sao Paulo	Promissão	SP_13C	21°32'S, 49°52'W		×	×		×	
*A. triste*	I	USNMENT01430154	Brazil	MGS	Brasilândia	MG_7A	21°16'S, 51°51'W	×	×	×	×	×	
*A. triste*	I	USNMENT01430155	Brazil	MGS	Brasilândia	MG_7B	21°16'S, 51°51'W	×	×	×	×	×	
*A. triste*	I	USNMENT01430156	Brazil	MGS	Brasilândia	MG_7C	21°16'S, 51°51'W		×	×	×	×	
*A. maculatum*	II		Colombia	Santander	Poima	SR_4A	6°09'N, 73°08'W		×	×		×	
*A. triste*	III	USNMENT01430102	Me×ico	Sonora	Mina Mulatos, Sahuaripa	M×_HO1	28°38'N, 108°44'W	×	×	×	×		
*A. triste*	III	USNMENT01430102	Me×ico	Sonora	Mina Mulatos, Sahuaripa	M×_HO2	28°38'N, 108°44'W	×	×	×	×		
*A. maculatum*	IV	USNMENT01430100	Perú	Ica	Ica	PU_IC10A	14°S, 75°46′W		×				
*A. maculatum*	IV	USNMENT01430101	Peru	Piura	Paimas	PU_PI1	4°37'S, 79°57´W	×	×	×		×	×
*A. maculatum*	IV	USNMENT01430101	Peru	Piura	Paimas	PU_PI2	4°37'S, 79°57'W	×	×	×		×	×
*A. triste*	IV		Peru	Tumbes	Pampas de Hospital	PU_TU1	3°41'S, 80°26'W	×	×	×		×	
*A. triste*	IV		Peru	Tumbes	Pampas de Hospital	PU_TU2	3°41'S, 80°26'W	×	×	×			
*A. triste*	IV		Peru	Tumbes	Pampas de Hospital	PU_TU3	3°41'S, 80°26'W	×	×	×	×	×	
*A. triste*	I		Uruguay	Canelones	EMA	CN_UY1	34°44'S, 55°58'W	×	×	×	×	×	
*A. triste*	I		Uruguay	Canelones	EMA	CN_UY2	34°44'S, 55°58'W	×	×		×	×	
*A. triste*	III	USNMENT00865800	USA	Arizona	Atascosa Spring, Santa Cruz Co.	AZ16_108	31°24'N, 111°10'W	×	×	×	×		×
*A. triste*	III	USNMENT00864570	USA	Arizona	Cave Canyon, Santa Cruz Co.	AZ16_14	31°43'N, 110°47'W	×	×	×	×	×	×
*A. triste*	III	USNMENT00865805	USA	Arizona	San Pedro Riparian NCA, Cochise Co.	AZ16_23	31°33'N, 110°08'W	×	×	×	×		
*A. triste*	III	USNMENT00865805	USA	Arizona	San Pedro Riparian NCA, Cochise Co.	AZ16_40	31°33'N, 110°08'W	×	×	×	×	×	×
*A. triste*	III	USNMENT00865805	USA	Arizona	San Pedro Riparian NCA, Cochise Co.	AZ16_121	31°22'N, 110°06'W	×	×	×	×	×	
*A. triste*	III	USNMENT00865801	USA	Arizona	Gardner Canyon, Santa Cruz Co.	AZ16_63	31°42'N, 110°47'W	×	×	×	×	×	×
*A. triste*	III	USNMENT00865800	USA	Arizona	Sycamore Canyon, Santa Cruz Co.	AZ16_1	31°25'N, 111°11'W	×	×		×		
*A. triste*	III	USNMENT00865800	USA	Arizona	Sycamore Canyon, Santa Cruz Co.	AZ16_4	31°25'N, 111°11'W	×	×	×	×		×
*A. maculatum*	II	USNMENT1430199	USA	Florida	Broward Co.	FL_24A	26°10'N, 80°51'W	×	×	×	×	×	×
*A. maculatum*	II	USNMENT1430194	USA	Georgia	Bulloch Co.	GA_19A	32°26'N, 81°51'W	×	×	×	×	×	
*A. maculatum*	II	USNMENT1430195	USA	Georgia	Bulloch Co.	GA_19B	32°26'N, 81°51'W	×				×	
*A. maculatum*	II	USNMENT1430196	USA	Georgia	Bulloch Co.	GA_20A	32°26'N, 81°51'W	×	×	×	×	×	
*A. maculatum*	II	USNMENT1430197	USA	Georgia	Bulloch Co.	GA_21A	32°26'N, 81°51'W	×	×	×	×	×	
*A. maculatum*	II	USNMENT1430198	USA	Georgia	Bulloch Co.	GA_21B	32°26'N, 81°51'W	×	×	×		×	
*A. maculatum*	II	USNMENT1430199	USA	Georgia	Bulloch Co.	GA_21C	32°26'N, 81°51'W	×	×	×	×	×	
*A. maculatum*	II	USNMENT1430200	USA	Georgia	Bulloch Co.	GA_21D	32°26'N, 81°51'W	×	×	×		×	
*A. maculatum*	II	USNMENT1430189	USA	Georgia	Camden Co.	GA_23A	30°58'N, 81°42'W	×	×	×	×	×	
*A. maculatum*	II	USNMENT1430184	USA	Georgia	Monroe Co.	GA_22A	33°02'N, 83°44'W	×	×	×	×	×	×

*Abbreviations*: *BA* Buenos Aires, *SDE* Santiago del Estero, *na* not applicable

### Common characters in males (Figs. 2, 3, 5, 6)

Body outline elongate-oval, narrower anterior to eyes; scapulae rounded; eyes flat. Scutum ornate, with reddish brown spots outlined by pale yellowish enameled stripes; postero-median spot extending anteriorly to level of spiracular plates; postero-accessory spots parallel to postero-median spot; three lateral spots large and sometimes fused; cervical spots narrow, first diverging, then slightly converging posteriorly; central area long, extending to mid-length of scutum. Chitinous scutes sometimes reduced to a tubercle, but always present on ventral surface of festoons. Basis capituli dorsally sub-rectangular, cornua long. Hypostome spatulate, dental formula 3:3. Legs: trochanters without spurs, coxa I with two distinct spurs, external spur long and sharp, internal spur as a small tubercle; coxae II-III with triangular, short, blunt spur each; coxa IV with narrow long sharp spur.

**Fig. 2:**
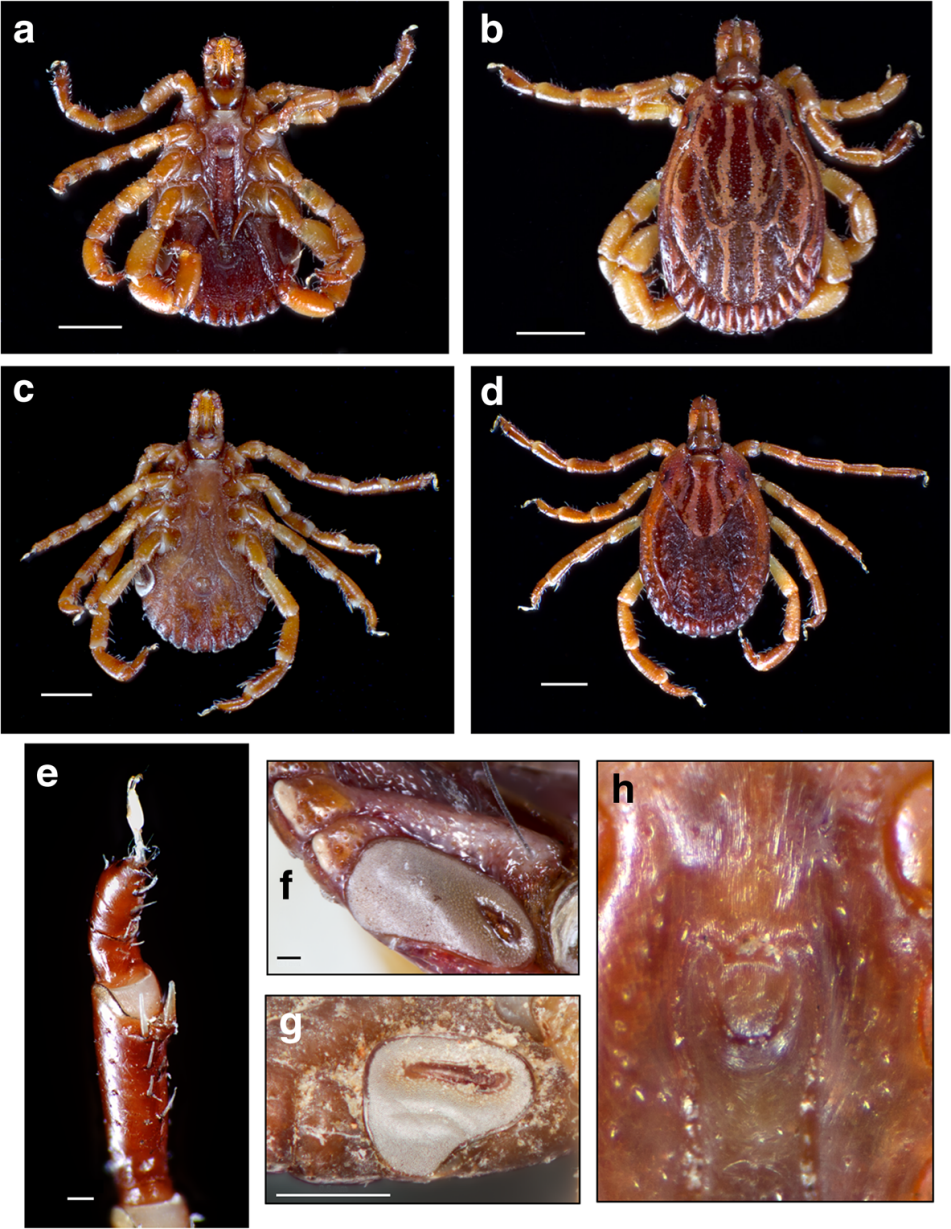
Morphotype I: *Amblyomma triste* (*sensu stricto*). Male, ventral (**a**) and dorsal (**b**) views; female, ventral (**c**) and dorsal (**d**) views; tibiae of legs II-IV (**e**); spiracular plates in the male (**f**) and female (**g**), and genital aperture (**h**). *Scale-bars*: **a**, 1 mm; **b**, 1 mm; **c**, 1 mm; **d**, 1 mm; **e**, 0.1 mm; **f**, 0.1 mm; **g**, 0.5 mm

**Fig. 3:**
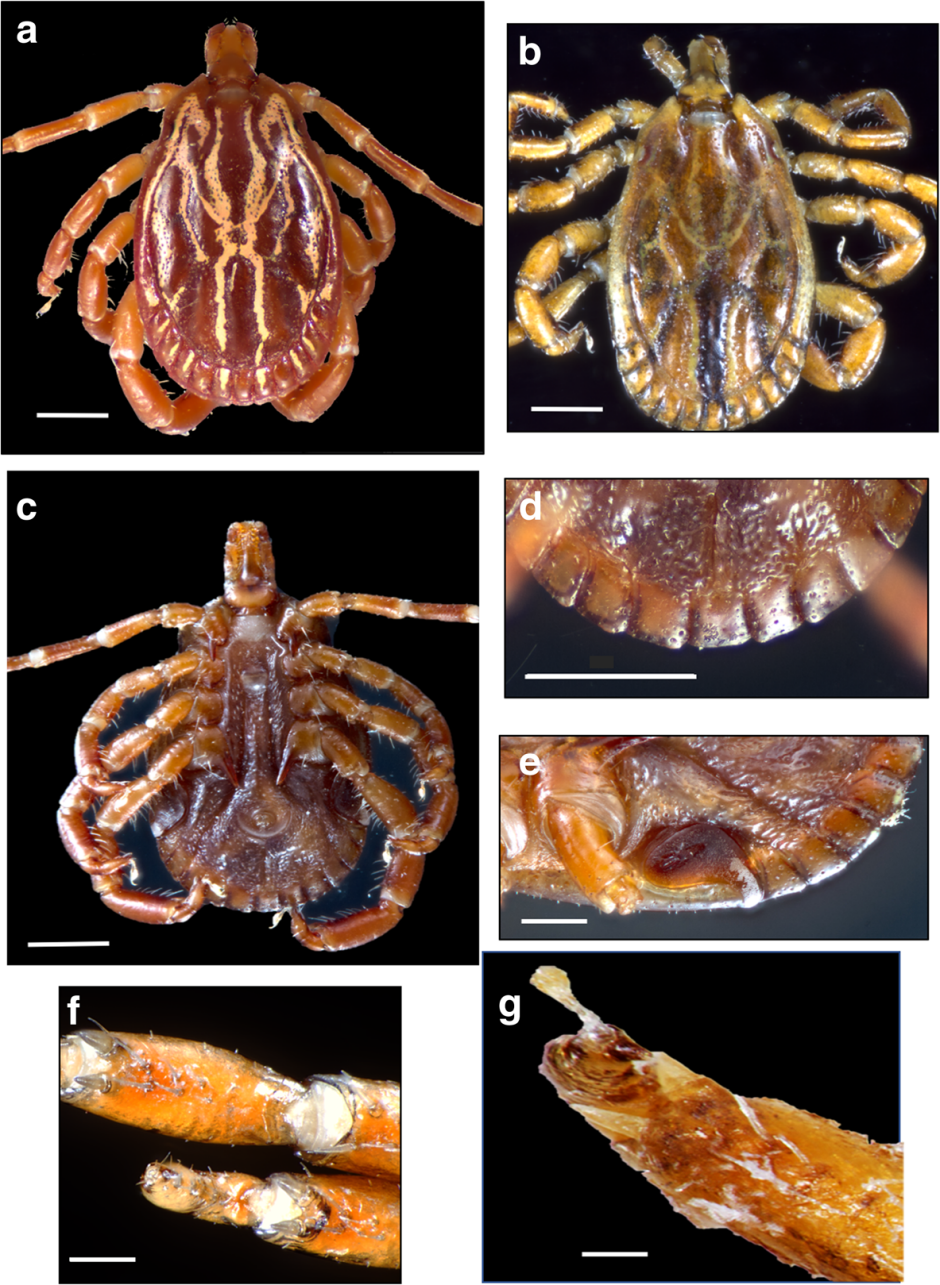
Morphotype II: *Amblyomma maculatum* (*sensu stricto*). Male, dorsal (**a**, USA; **b**, Venezuela) and ventral (**c**, **d**, USA) views; male, spiracular plates (**e**, USA); tibiae of legs II-IV in the male (**f**, USA) and female (**g**, USA). *Scale-bars*: **a**, 1 mm; **b**, 1 mm; **c**, 1 mm; **d**, 1 mm; **e**, 0.5 mm; **f**, 0.1 mm; **g**, 0.1 mm

### Common characters in females (Figs. 2–6)

**Fig. 4:**
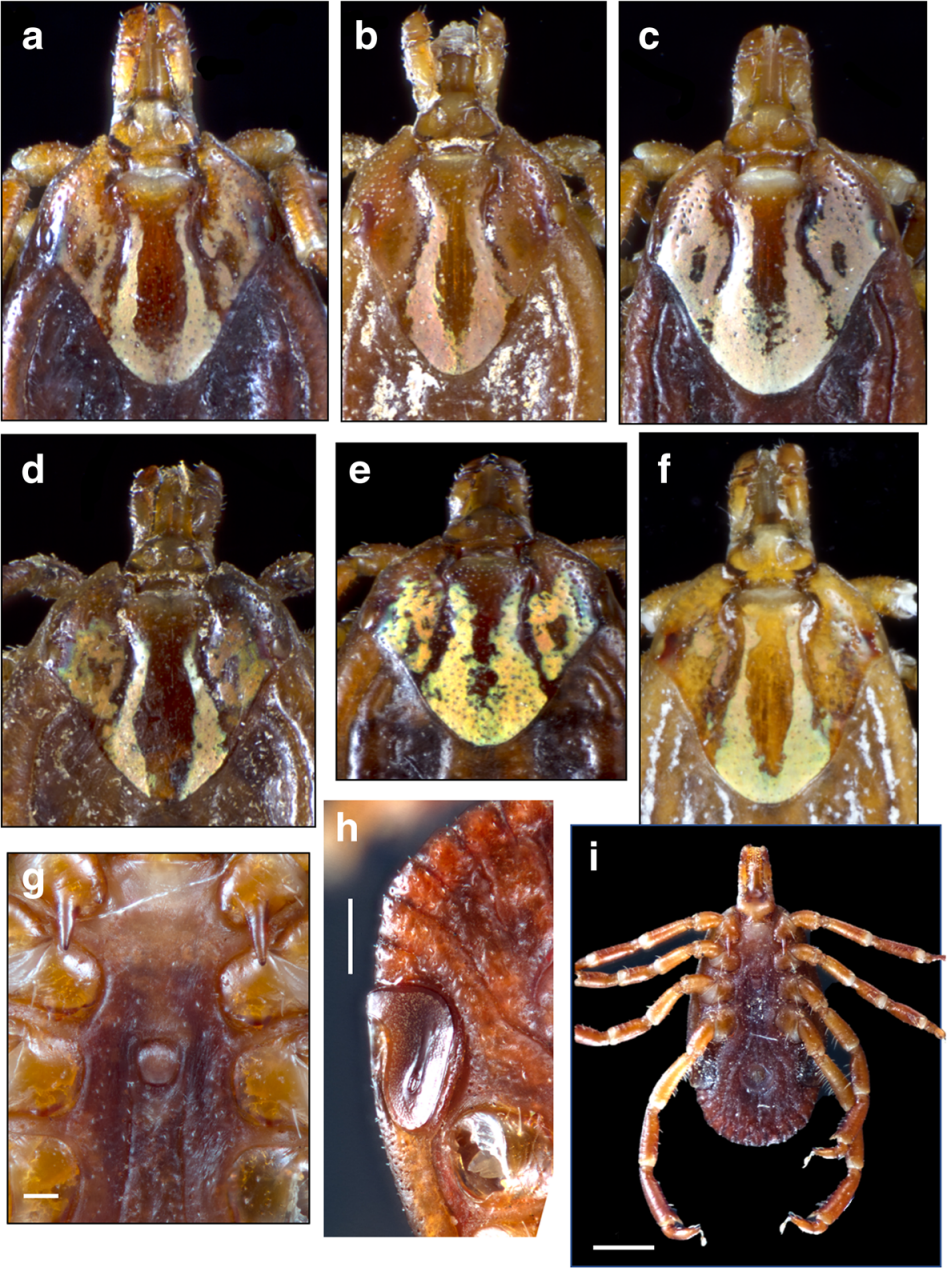
Morphotype II: *Amblyomma maculatum* (*sensu stricto*). Female, variation in dorsal ornamentation (**a-e**, USA; **f**, Venezuela); genital aperture (**g**, USA). Male, spiracular plates (**h**, USA); male, ventral view (**i**, USA). Scale-bars: **g**, 0.2 mm; **h**, 0.5 mm; **i**, 1 mm

**Fig. 5:**
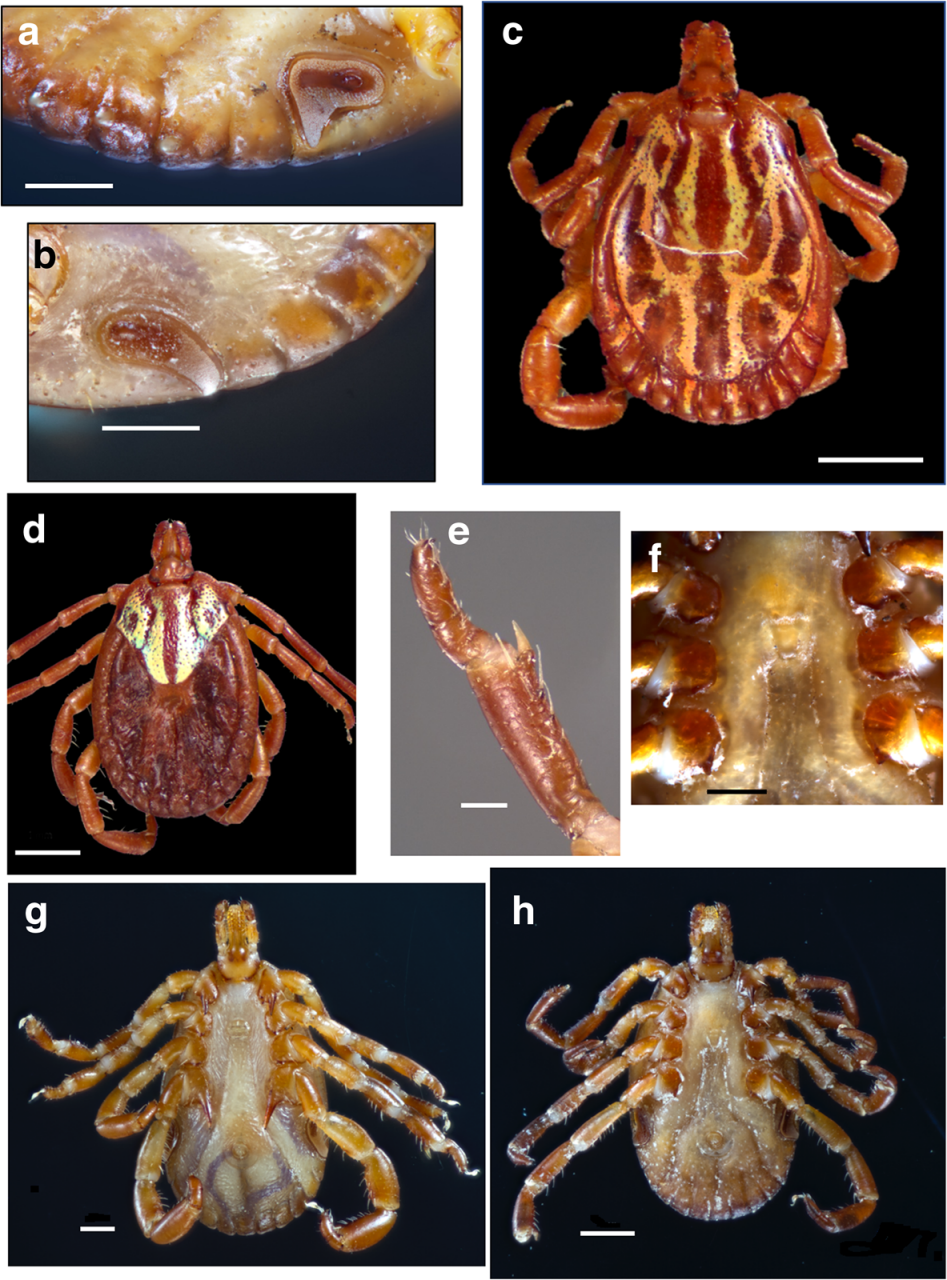
Morphotype III: *Amblyomma* sp. cf. *A. triste - A. maculatum* [Arizona (USA) and Mexico]. Female, spiracular plate (**a**); male, spiracular plate (**b**); male, dorsal view (**c**); female, dorsal view (**d**); tibiae legs II-IV (**e**); female, genital aperture (**f**); male, ventral view (**g**); female, ventral view (**h**). *Scale-bars*: **a**, 0.5 mm; **b**, 0.5 mm; **c**, 1 mm; **d**, 1 mm; **e**, 0.1 mm; **f**, 0.5 mm; **g**, 0.5 mm; **h**, 1 mm

**Fig. 6:**
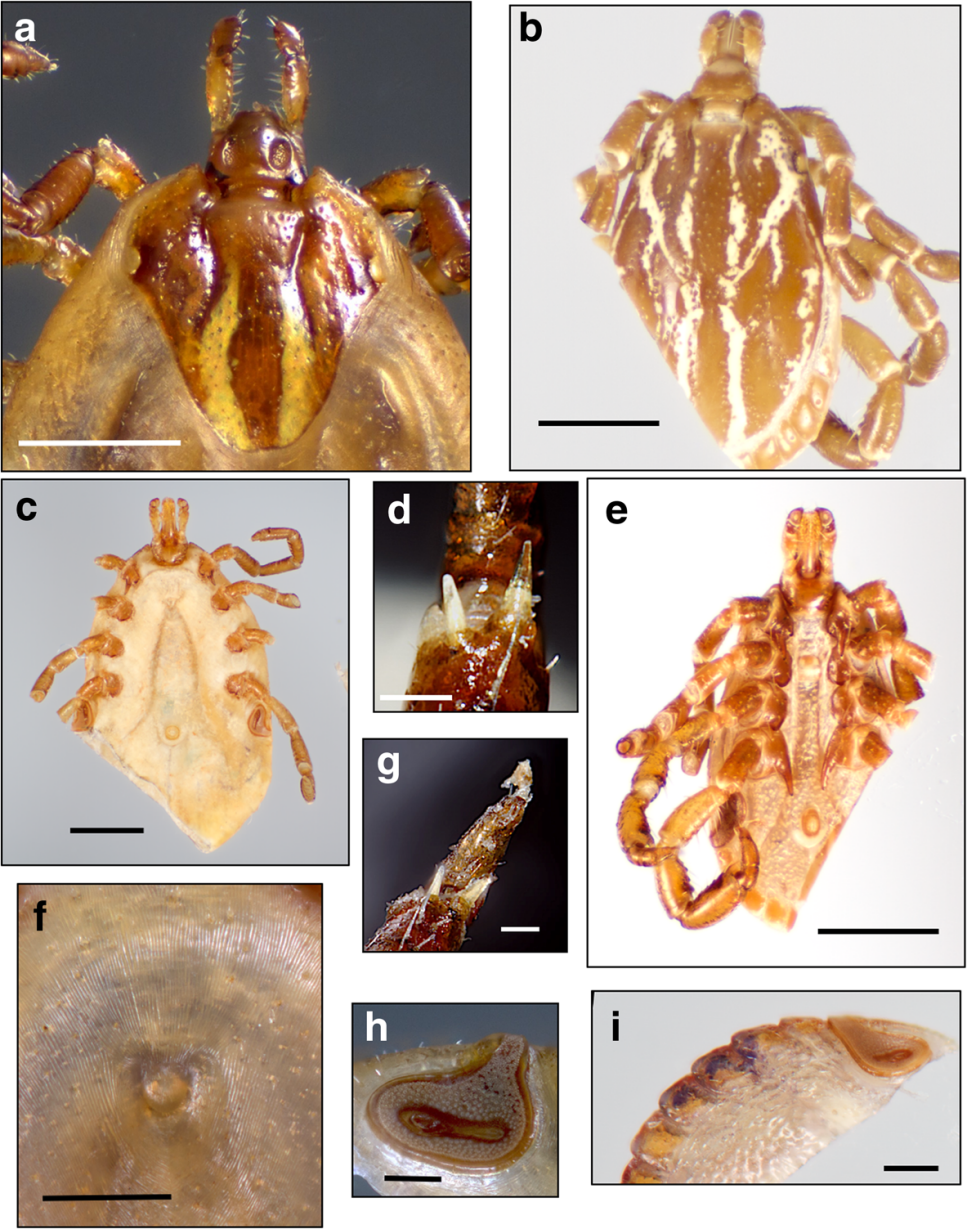
Morphotype IV: (Peru) *Amblyomma* sp. cf. *A. triste - A. maculatum* (Peru). Female, scutum (**a**); male, dorsal view (**b**); tibiae of legs II-IV (**d**, **g**); male, ventral view (**e**); female, genital aperture (**f**); female, spiracular plates (**h**, **i**). **a**, 1 mm; **b**, 1 mm; **c**, 1 mm; **d**, 0.1 mm; **e**, 1 mm; **f**, 0.5 mm; **g**, 0.1 mm; **h**, 0.5 mm, **i**, 0.5 mm

Body outline oval. Scapulae rounded, cervical grooves deep anteriorly, shallow posteriorly, sigmoid in shape; eyes flat. Small chitinous tubercles dorsally visible at postero-medial edge of festoons. Scutum ornate, extensively pale yellowish, cervical spots narrow and divergent posteriorly, brown central area long, narrow. Notum glabrous dorsally, but with fine, very short, evenly distributed setae ventrally. Basis capituli dorsally sub-rectangular, cornua absent, porose areas oval. Hypostome spatulate, dental formula 3:3. Legs: coxa I with two distinct spurs, external spur long and sharp, internal spur as a small tubercle; coxae II-IV each with a triangular, short blunt spur; trochanters without spurs. Genital aperture U-shaped (Figs. 2h, 4g, 5f, 6f).

### Morphotype I: *Amblyomma triste* Koch, 1844 (*sensu stricto*)

***Distribution*:** Present study: Argentina, Paraguay, southern Brazil and Uruguay.

***Type- and voucher material examined*:** Type-specimens of *A. triste* (ZMB 1046): 2♀♀, Montevideo, Uruguay. Voucher specimens: *Argentina*: Buenos Aires Province: 5♀♀ 5♂♂, Reserva Otamendi (INTA 1978); 30♀♀ 30♂♂, Inta Delta del Paraná (INTA 1979, 2009, 2033, 2042, 2065, 2104, 2115, 2198); 1♀ ♂, Punta Indio (INTA 2225); 1♂, San Nicolás (INTA 2184); 1♀ 1♂ Dársena Paraná Guazú (INTA 2094); Corrientes Province: 1♀ (USNMENT00958436); 1♀ Estero del Iberá (INTA 1914); 1♀ 1♂, Estancia Rincón del Socorro (INTA 2129); Entre Ríos Province: 1♂, Villa Paranacito (INTA 2194); 1♀ 1♂, Isla Paloma (INTA 2114); Formosa Province: 1♀ 1♂, Reserva El Bagual (INTA 2096); 1♀ Colonia Pastoril (INTA 2151). *Bolivia*: Beni Department, 1♀, Pampa de Mais (USNMENT00958430). *Brazil*: State of Mato Grosso do Sul: 1♀ 1♂, Bataguassu (INTA 1803); 2♀♀ 2♂♂, Agua Clara (USNMENT01430164); State of São Paulo: 3♀♀ 3♂♂, Promissão (USNMENT01430159); 1♀, Porto Primavera (USNMENT00958438). *Paraguay*: locality not indicated, 1♀ 1♂, (USNMENT00958439); Boquerón: 3♂♂ Estancia Faro Moro (USNMENT00958431). *Uruguay*: Canelones Department: 46♀♀ 18♂♂, Toledo Chico (DPVURU 104); 54♀♀ 28♂♂, Progreso (DPVURU 149); Montevideo Department: 45♀♀ 33♂♂, Villa García, (DPVURU 181); San José Department: 5♀♀ 2♂♂, Playa Pascual (DPVURU 117).

### Description (Fig. 2a-h)

***Male.*** Chitinous scutes on ventral surface of festoons well developed (Fig. 2a), visible dorsally (Fig. 2b), sometimes lined with whitish enamel (Fig. 2c, f). Postero-median spot wider than enameled stripe between postero-median and postero-lateral spots (Fig. 2b). One spine and one fine seta present on tibiae of legs II-IV (Fig. 2e). Spiracular plates almost oval, with short, wide dorsal projection (Fig. 2f), as wide as adjoining festoon, spurs on coxa IV long and sometimes reaching anus (Fig. 2a).

***Female.*** Brown central spot extending to the posterior margin of the scutum present (Fig. 2d). One spine and one seta on tibiae of legs II-IV present as in male. Spiracular plates usually oval, with short, wide dorsal projection longer than in males, dorsal projection as wide as adjoining festoon (Fig. 2g).

### Morphotype II: *Amblyomma maculatum* Koch, 1844 (*sensu stricto*)

***Distribution*:** Present study: Colombia, Guatemala, Honduras, Mexico, Nicaragua, USA and Venezuela.

***Type- and voucher material examined*:** Type-specimen of *A. maculatum* (ZMB 1044): 1♂, “Carolina”, USA (presumably from one of the Carolinas). *Colombia*: Chachagüí Department: 3♀♀1♂, Pasto (USNMENT00957339); 3♀♀ 12♂♂, La Florida (USNMENT00957340, 00957359, 00957329); Valle del Cauca Department: 6♀♀ 3♂♂ Cali (USNMENT00957349, 00957344, 00957342, 00957325. *Guatemala*: Chimaltenango Department: 1♂, Acatenango (USNMENT00957404). *Honduras*: Atlántida Department: 1♀, Camp Dakota (USNMENT00957346); 6♂♂, locality not specified (USNMENT957405). *Mexico*: Sinaloa Department: 1♀, Culiacan (USNMENT00957402); 5♀♀ 7♂♂ Los Mochis (USNMENT00957387, 00957378). *Nicaragua*: Carazo Department: 1♂, Dolores (USNMENT00957406); Rivas Department: 1♂, Rivas (USNMENT957408). *USA*: Alabama: 1♂, Sumter Co. (USNMENT00957286), 1♂, Houston Co. Dothan Landmark Park (USNMENT00957256); Florida: 1♀ 2♂♂, Orange Co, Orlando (USNMENT00957220), 3♀♀ 1♂, Palm Beach (USNMENT00957250), 2♂♂, Marion Co, Ocala (USNMENT00957350), 1♀ 7♂♂, Collier Co, Bear Island (USNMENT957234), 1♀ 1♂, Golden Gate (USNMENT957304); Georgia: 3♀♀ 1♂, Grady Co, Cairo, (USNMENT00957242); 2♀♀ 4♂♂, Thomas Co, Thomasville (USNMENT00957384), 1♂, Bulloch Co, Statesboro (USNMENT00957364), 3♀♀, Decatur Co, Bainbridge (USNMENT00957290), 1♀, Lowndes Co. Moodyfields Airforce Base (USNMENT00957310), 1♀, Liberty Co, St. Catherine Isl. (USNMENT00957334); Indiana: 1♂, Hamilton Co, Carmel, (USNMENT00957317); Louisiana: 4♀♀ 1♂, Cameron Co, Johnson’s Bayou (USNMENT00957271), 1♀ 2♂♂, Cameron (USNMENT00957280), 1♀ 2♂, Baton Rouge (USNMENT00957373, 00957353); Mississippi: 2♂♂ Pearl River Co, Mc Neill (USNMENT00957372), 3♀♀, Poplarville (USNMENT00957273), 1♂, Prentiss Co, Booneville (USNMENT00957331), 1♀ 2♂♂, Copiah Co, Copiah (USNMENT00863742, 00957332), 1♂, Covington Co, Collins (USNMENT00957281), 1♀ Hinds Co, Jackson (USNMENT00957334), 2♀♀, Forest Co. (USNMENT00957302); Oklahoma: 1♀, Pittsburg Co, Weathers (USNMENT00957366), 2♀♀, Mayes Co, Chouteau (USNMENT00957356), 2♂♂ 2♀♀, Osage Co. (USNMENT00957292). State of South Carolina: 3♂♂ 1♀, Charleston Co, Charleston (USNMENT00957380), 6♀♀, Johns Isl. (USNMENT00957283), 1♂ 1♀, Allendale Co, Allendale (USNMENT00957266); 2♀♀, Georgetown Co, Georgetown (USNMENT00957270), 1♂, Jasper Co, Ridgeland (USNMENT00957246). Texas: 2♂, Dallas (USNMENT00957239, 00957385), 17♀♀, Galveston Co, High Island (USNMENT00957263, 00957313), 2♂♂ 1♀, Austin Co, Sealy (USNMENT00957232), 35♂♂ 9♀♀, Cat Springs (USNMENT00863706, 00957375, 00957370), 2♂♂ New Ulm (USNMENT00957351), 1♂ 1♀, Jackson Co. (no locality) (USNMENT00957240), 5♂♂ 5♀♀, Victoria Co. Victoria (USNMENT00957237, 00957377), 2♀♀, Nueces Co, Corpus Christi (USNMENT00957382), 5♂♂ 1♀, Fayette Co, Shulenberg (USNMENT00957381), 16♂♂ 6♀♀, Jim Hogg Co, Hebbronville (USNMENT00957288). *Venezuela*: State of Cojedes: 2♂♂, Cojedes (USNMENT00957347); State of Miranda: 1♀, Alto de Pipe (USNMENT00957362); State of Monagas: 2♀♀ 1♂, San Agustin (USNMENT00957337, 00957333).

### Description (Figs. 3a-g, 4a-i)

***Male.*** Chitinous scutes present on ventral surface of festoons, often less salient than in Morphotype I, sometimes reduced to a posteromedian tubercle (Fig. 3c-e). Two robust spines almost equal in size present on tibiae of legs II-IV (Fig. 3f). Spiracular plates comma-shaped, with tip of dorsal projection equal in width or slightly narrower than half of adjacent festoon; dorsal projection perpendicular to longitudinal axis of spiracular plate (Fig. 3e). Spurs on coxa IV not reaching anus (Fig. 3c); postero-median spot wider than enameled stripe between postero-median and postero-lateral spots (Fig. 3a, b).

***Female.*** Two spines almost equal in size present on tibiae of legs II-IV (Fig. 3g). Spiracular plates comma-shaped, with dorsal projection perpendicular to axis of spiracular plate, as wide or slightly wider than half-festoon width (Fig. 4h). Scutal brown central area long and narrow, not reaching posterior margin of scutum in most specimens, a few specimens with central area reaching posterior margin of the scutum; enameled surface of scutum variable in shape and proportions (Fig. 4a-f).

### Morphotype III: *Amblyomma* sp. cf. *A. triste* - *A. maculatum*

***Distribution*:** Present study: northern Mexico and Arizona, USA.

***Voucher material examined*:** *Mexico*: State of Sonora, Hermosillo, Minas Mulatos 8♀♀ 6♂♂, (USNMENT01430102), 1♂, Locality not specified (USNMENT958432). *USA*: Arizona: 1♀ 1♂, Cochise Co, San Pedro Riparian National Conservation Area (USNMENT00865805), 1♀ 1♂, Santa Cruz Co. Santa Rita Mountains, Gardner Canyon (USNMENT00865801), 1♀ 1♂, Santa Cruz Co, Pajarita Wilderness Area (USNMENT00865800), 1♀ 1♂, Santa Cruz Co, Santa Rita Mountains, Cave Canyon (USNMENT00864570), 1♀ 1♂, Cochise Co, San Pedro Riparian Conservation Area, Herford Road (USNMENT00864571) and all ticks used in [23].

### Description (Fig. 5a-h)

***Male.*** Chitinous scutes present on ventral surface of festoons (Fig. 5g), visible dorsally (Fig. 5a-c). Postero-median spot wider than enameled stripe between postero-median and postero-lateral spots in most specimens (Fig. 5c), a few specimens with postero-median spot almost equal in width to stripe between postero-median and postero-lateral spots. One spine and one seta present on tibiae of legs II-IV (Fig. 5e). Spiracular plates comma-shaped, with dorsal projection approximately half as wide as adjoining festoon; dorsal projection as an extension of spiracular plate, not perpendicular to its axis (Fig. 5b). Spurs on coxa IV not reaching anus (Fig. 5g).

***Female.*** Brown scutal central area long and narrow, always reaching posterior margin of scutum (Fig. 5d). One spine and one seta present on tibiae of legs II-IV as in male. Spiracular plates with dorsal projection perpendicular to plate axis, as wide or narrower than a third of adjoining festoon (Fig. 5a).

### Morphotype IV: *Amblyomma* sp. cf. *A. triste* - *A. maculatum*

***Distribution*:** Present study: Chile, Ecuador and Peru.

***Voucher material examined*:** *Chile*: XV Arica and Parinacota Region: 1♀ 1♂, Arica (CNC 1918). *Ecuador*: Los Ríos Province: 1♂, locality not indicated (USNMENT00958433); Guayas Province: 1♂, “litoral region” (USNMENT00958435); 1♂, Naranja (USNMENT00958428); El Oro Province: 1♀, Machala (USNMENT00957381). *Peru*: Ica Department: 1♀, Ica (USNMENT01430100); Piura Deparment: 1♀ 1♂, Piura (USNMENT01430101). Images taken after DNA was extracted from specimens.

### Description (Fig. 6a-i)

***Male.*** Chitinous scutes present on ventral surface of festoons (Fig. 6e), visible dorsally (Fig. 6b). Two spines slightly different in size present on tibiae of legs II-IV but originating at a different level along tarsi and therefore appearing to be of different lengths (Fig. 6d). Spiracular plates comma-shaped, with dorsal projection about half as wide as adjoining festoon extending from plate at an obtuse angle (Fig. 6i). Postero-median spot wider than enameled stripe between postero-median and postero-lateral spots (Fig. 6b).

***Female.*** Central scutal area long, narrow (Fig. 6a), reaching posterior margin of scutum in most specimens. Spines on tibiae of legs II-IV as in males (Fig. 6g). Spiracular plates comma-shaped, with dorsal projection perpendicular to plates and approximately half as wide as adjoining festoon.

### PCR amplification and sequence alignment

Although we did not obtain all gene sequences for each sample, we obtained sequences representative for all species and morphotypes (Table 1). The lengths of the data matrices were as follows: 338 bp for *12S* rDNA, 410 bp for *16S* rDNA, 497 bp for *cox*1, 368 bp for DL, and 954 bp for ITS2. The concatenated mitochondrial dataset (mtDNA) included 1604 bp, the mt+nDNA 2556 bp, and the alignment for molecular clock and dating analyses 1469 bp (after removing regions that were too variable between the *A. maculatum* and the *A. cajennense* groups for unambiguous alignment). GenBank accession numbers for sequences generated in the present study are: KU284849-KU284860, MG076929-MG076938 (*A. maculatum 12S* rDNA); KU284861, KU284862 (*A. neumanni 12S* rDNA); KU284863, KU284864 (*A. parvitarsum 12S* rDNA); KU284865-KU284921 (*A. triste 12S* rDNA); KU284922-KU284929 (*A. tigrinum 12S* rDNA); KU284930-KU284941, KU284999, MG076911-MG076919 (*A. maculatum 16S* rDNA); KU284942-U284998, KU285000, KU285001 (*A. triste 16S* rDNA); KU285002-KU285010 (*A. tigrinum 16S* rDNA); KU285011 (*A. neumanni 16S* rDNA); KU285012 (*A. parvitarsum 16S* rDNA); KU285013-KU285024 (*A. maculatum* DL); KU285025-KU285079 (*A. triste* DL); KU285080-KU285086 (*A. tigrinum* DL); KU285087 (*A. neumanni* DL); KU285088-KU285094, MG076920-MG076928 (*A. maculatum* ITS2); KU285095-KU285137 (*A. triste* ITS2); KU285138, KU285139 (*A. tigrinum* ITS2); KU306550-KU306598 (*A. triste cox*1); KU306599 (*A. neumanni cox*1); KU306600 (*A. parvitarsum cox*1); KU302492-KU302504, MG251313 (*A. maculatum cox*1); KU302505-KU302511 (*A. tigrinum cox*1); KU306601-KU306604 (*A. maculatum cox*2); KU306605-KU306607 (*A. tigrinum cox*2); KU306608-KU306611 (*A. triste cox*2); KU306612 (*A. parvitarsum*); KU306613 (*A. neumanni cox*2). Figure 1 illustrates the sites where the ticks used for phylogenetic analyses were collected.

### Sequences and haplotype diversity

If we exclude the outgroup sequences, the 87 *12S* rDNA gene sequences were represented by 17 unique haplotypes (Additional file 1: Table S1), the 91 *16S* rDNA sequences by 34 unique haplotypes (Additional file 2: Table S2), the 73 *cox*1 sequences by 32 unique haplotypes (Additional file 3: Table S3), the 81 DL sequences by 40 unique haplotypes (Additional file 4: Table S4) and the 62 ITS2 sequences by 15 unique genotypes (Additional file 5: Table S5). A total of 42 haplotypes were unique among the 63 concatenated mtDNA sequences, and 27 among the 31 concatenated mt+nDNA datasets. For all mitochondrial datasets, haplotypes were consistently shared by ticks corresponding to Morphotypes II and III. Otherwise, no haplotypes were shared between other morphotypes.

### Phylogenetic analyses

#### Separate datasets

The separate gene sequence analyses (phylogenies not shown) were characterized by overall limited resolution. The ingroup was consistently strongly supported. *Amblyomma tigrinum* was always monophyletic, mostly as the sister lineage to the *A. maculatum - A. triste* cluster (*16S* rDNA, DL and ITS2), but also as the sister group of Peruvian Morphotype IV (*cox*1) or embedded within Morphotype I (*12S* rDNA). The overall structure of the five phylogenies was different, with Morphotypes II and III never clustering in two separate lineages, while the southern South American and the Peruvian sequences were sometimes identifiable as distinct clades (*cox*1). In addition, the analysis of ITS2 sequences only identified a supported polytomic ingroup. A closer examination of the ITS2 sequences revealed that when the matrix included outgroups, informative characters were 108 (11%); however, when outgroup sequences were excluded, informative characters only represented 0.9%. In the ITS2 ingroup matrix substitutions were mostly randomly scattered singleton mutations and indels with no phylogenetic information.

#### Mitochondrial concatenated datasets (*12S* rDNA + *16S* rDNA + *cox*1 + DL)

**Fig. 7:**
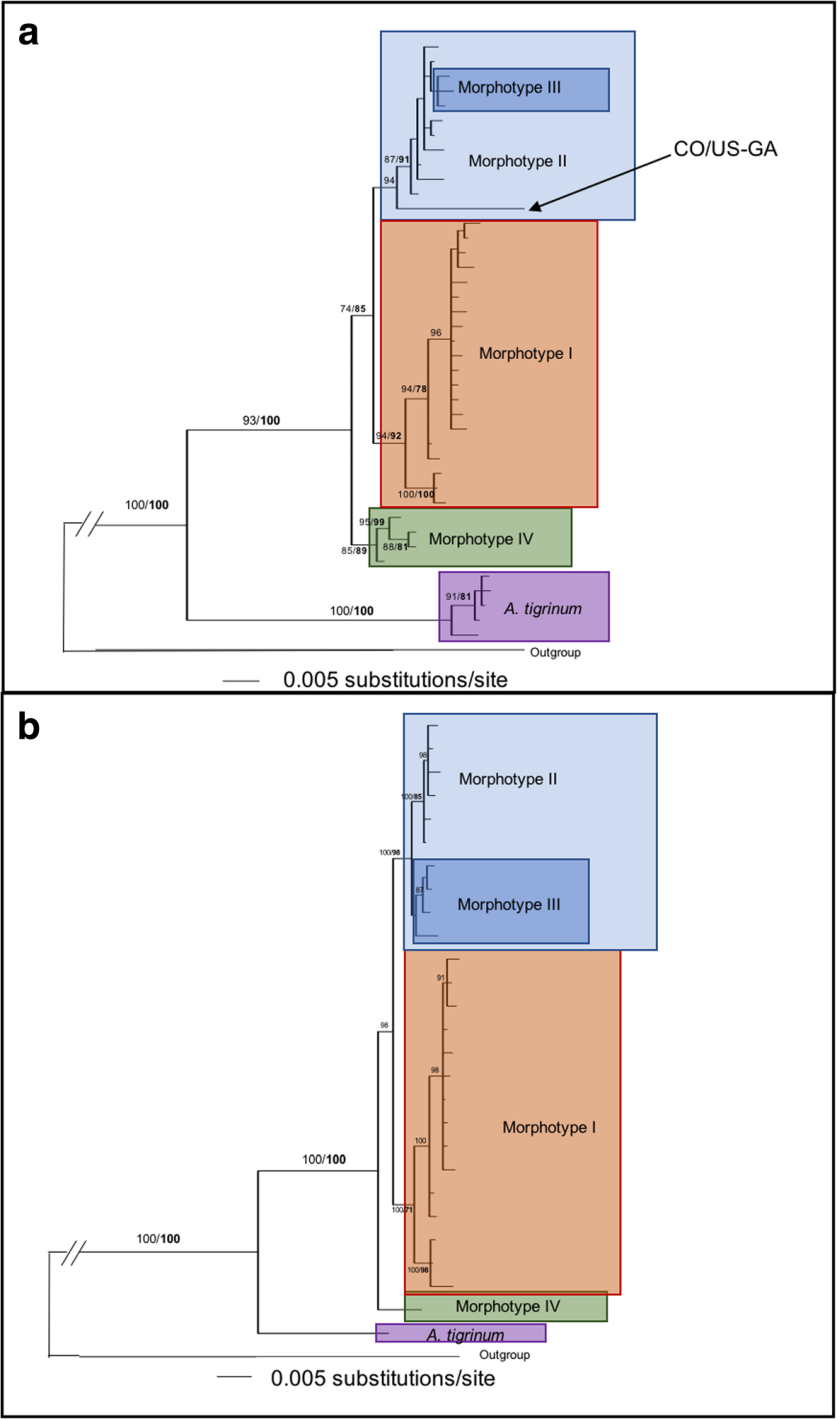
Phylogenetic reconstruction of the mitochondrial concatenated dataset (**a**), and the total evidence (mitochondrial + nuclear) concatenated dataset (**b**). Trees were inferred by Bayesian analysis. Numbers on branches represent maximum parsimony bootstrap support and Bayesian posterior probability support (in bold)

The MP (Maximum Parsimony) analysis of the mtDNA matrix found 6586 equally parsimonious trees (length = 506). The resolution of the MP strict consensus tree was better than that recovered for the separate datasets. The *A. tigrinum* and the *A. maculatum - A. triste* clusters were sister clades and both were well supported. Within the *A. maculatum - A. triste* clade, the first split occurred between Morphotype IV (Peru) and the remaining lineages. Morphotypes II and III were intermixed within a single clade, except for one sequence (identified as CO-US/GA in the tree) which corresponded to a Colombian sequence. Morphotype I was sister to Morphotypes II+III and included all southern South American sequences. Bayesian analysis recovered an almost identical topology (Fig. 7). Within these supported clades, resolution was very limited and when monophyletic, clades did not correspond to any geographical or ecologically meaningful subset.

#### Mitochondrial-nuclear concatenated dataset (*12S* rDNA + *16S* rDNA + *cox*1 + DL + ITS2)

The concatenated dataset represented a matrix of 2556 bp (31 sequences, corresponding to 27 unique haplotypes and one outgroup). The MP analysis identified a total of 56 equally parsimonious trees (length = 770). The MP and Bayesian inference BI reconstructions were almost completely congruent: the ingroup was monophyletic and *A. tigrinum* was consistently identified as the supported sister clade to the monophyletic *A. maculatum - A. triste* clade (Fig. 7b). However, by MP, the *A. maculatum - A. triste* clade was polytomic leading to monophyletic Morphotypes I, II+III, and IV. While Morphotype II was found to be monophyletic, Morphotype III did not cluster in a single supported clade. By BI, Morphotype IV was found to be the sister clade to the remaining morphotypes which clustered in a well-supported lineage (Fig. 7b). Morphotype I was monophyletic and, again, the structure within Morphotype I did not appear to correspond to geographical subdivisions with sequences from different regions represented in each clade. The last monophyletic clade included a monophyletic Morphotype II and a paraphyletic Morphotype III (Fig. 7b).

Divergence values in the mt+nDNA dataset, calculated after the best mutation model was evaluated by ModelTest, showed that the distance between outgroup and ingroup varied from 18.60 to 19.01%, between *A. tigrinum* and *A. maculatum - A. triste* by 3.91–4.31%, between Morphotype IV and the remaining clades by 1.22–1.70%, between Morphotype I and II+III by 0.91–1.22% and between Morphotype II and III by 0.32–0.71%. Within Morphotype II, distances varied between 0 and 0.28%, within Morphotype III between 0 and 0.51% and within Morphotype I between 1.22 and 1.70%.

### Node dating

**Fig. 8:**
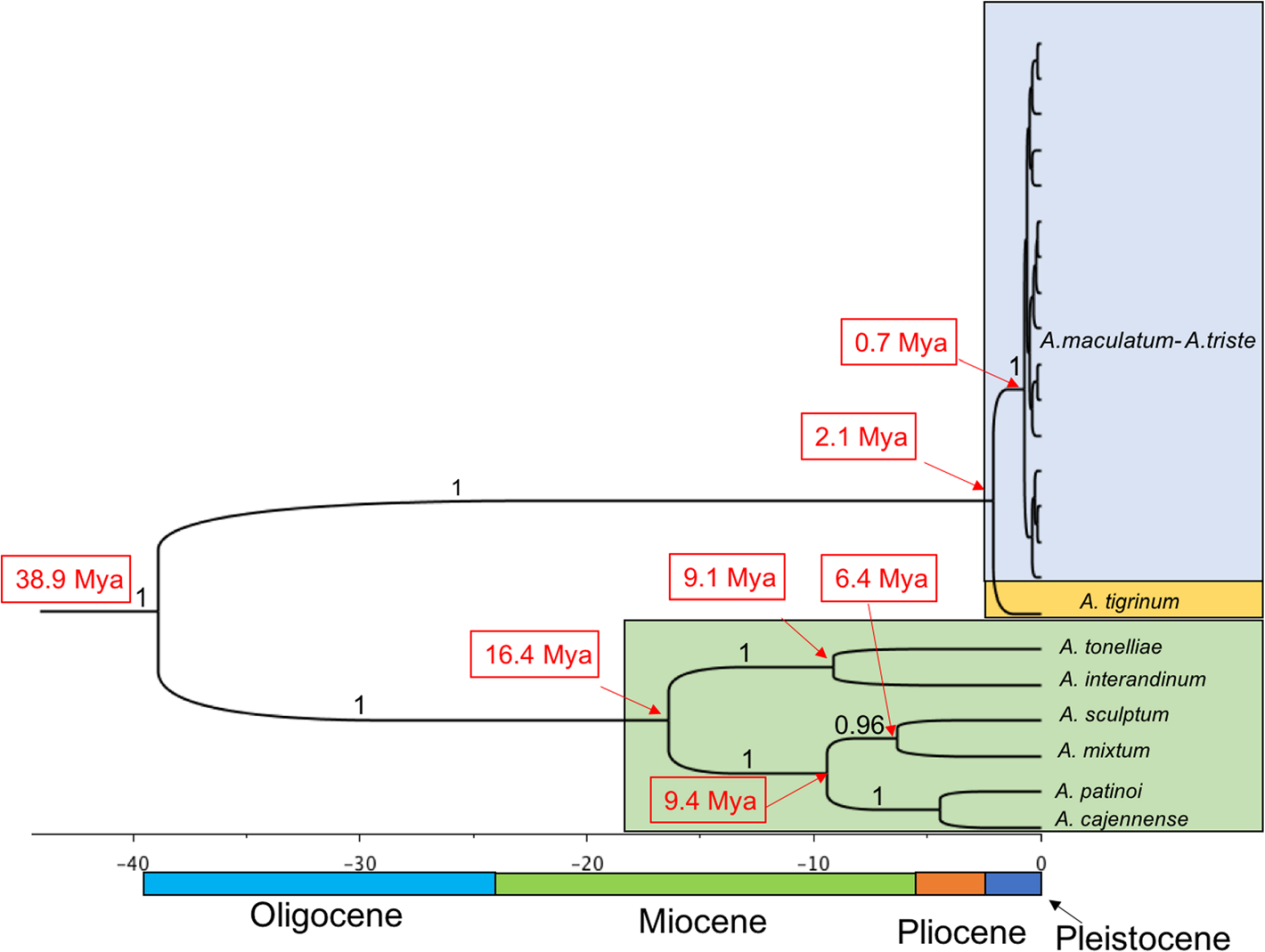
Tentative node dating of the main nodes on a tree generated by BEAST using the relaxed clock method. Numbers (in black) over branches represent posterior probability support, while the dates are represented in red and in millions of years ago (Mya)

DAMBE analyses revealed no significant mutation rate differences between the main ingroup lineages (*P =* 0.053–0.9) including *A. tigrinum*. Both the least-square method and the likelihood ratio test did not reject the molecular clock hypothesis, further confirming that the lineages under consideration did not evolve at significantly different rates. Although this would justify the use of a strict molecular clock in BEAST, we selected the relaxed molecular clock option which allows for variable mutation rates because mutation rates differed significantly between ingroup and outgroup (*P* ≤ 0.01). The tree generated by BEAST supported the basal subdivision between *A. tigrinum* and *A. maculatum - A. triste*, but did not resolve relationships within the *A. maculatum - A. triste* polytomic clade (Fig. 8). By using a single calibration point, we first confirmed that the nodes within the *A. cajennense* group were consistent with prior findings [36] associating nodes with known biogeographical vicariant events. The diversification of the *A. maculatum* group of taxa appeared to be more recent with the split between *A. tigrinum* and the *A. maculatum - A. triste* clades dating back to approximately 2.1 Mya (95% confidence interval: 0.9–3.3 Mya) and the *A. maculatum - A. triste* radiation beginning no sooner than 720,000 years ago (95% confidence interval: 0.3–1.2 Mya) in the mid-Pleistocene (Fig. 8). The split between the outgroup and the ingroup dated was estimated at 39 Mya (95% confidence interval: 14–70 Mya).

## Discussion

Morphological reassessment of the *A. maculatum - A. triste* specimens revealed four morphotypes distinguishable by four distinct sets of characters. Morphotype I includes specimens belonging to populations from Argentina, Paraguay, Uruguay and southern Brazil. These ticks were assigned to the taxon *A. triste* (*s.s.*) because their morphology is compatible with the morphology of the type-specimens of *A. triste* (ZMB 1046) from Montevideo, Uruguay (see [4]). Morphotype II includes specimens from eastern USA, Colombia, Guatemala, Honduras, Mexico and Venezuela, with a morphology matching the type-specimens of *A. maculatum* (*s.s.*) (ZMB 1044) also described by Koch [4] from a type-locality reported as “Carolina”, USA (possibly in one of the Carolina States which are included in the present distribution of *A. maculatum*). Morphotypes I and II have so far been differentiated by examining the tibial armature of legs II-IV, the shape of the male spiracular plates and, in some cases, scutal ornamentation in females. The remaining two morphotypes present a combination of characters that precludes a strict assignation to either *A. triste* or *A. maculatum*, which explain why Peruvian and Chilean samples have alternatively been considered to be *A. maculatum* or *A. triste* [10, 37, 38], and Arizona samples have been assigned to *A. triste* [11], based on tibial armature morphology. Morphotype III includes ticks from the southwestern USA (Texas and Arizona) and northern Mexico. It could also correspond to what Guzman-Cornejo et al. [22] described as *A. triste* from Mexico, although the specimens should be reexamined. They have one spine and one seta on tibiae of legs II-IV, comma-shaped spiracular plates (elongated in males), and the scutum of females with a central spot reaching the posterior margin of the scutum. Morphotypes III and IV are similar, but Morphotype IV is characterized by the presence of two spines which appear to be of different length on tibiae of legs II-IV. However, when these phenotypic differences are closely scrutinized, with the exceptions of the overall shape of the spiracular plates and differences in thickness of the tibial setae on legs II-IV, none of the other phenotypic features appear to be fixed when sufficient numbers of ticks from each area are observed. All other differentiating features are shared by at least two of the four morphotypes (Table 2). This situation raises the question of whether we are dealing with four different species, less than four, or a single species.

**Table 2:** Summary of distinctive morphological character combinations in the four different morphotypes

Morphotype	Origin	Males	Males	Males + Females	Males	Females	Females
		Scutes on ventral festoons	Postero-median spot in relation to width of adjacent stripe	Tibial armature	Spiracular plates (width of projection in relation to width of adjacent festoon)	Spiracular plates (width of projection in relation to width of adjacent festoon)	Brown central spot
I	UY, AR, BR	Present	Wider	1 spine + 1 stout seta	Oval, with short wide projection (± 1 festoon)	Comma-shaped, wide (± 1 festoon)	Reaching posterior margin of scutum
II	USA, CO, CR, VE	Present, less conspicuous	Wider	2 spines of almost equal length	Comma-shaped with narrow projection (± 1/2 festoon)	Comma-shaped, with narrow projection (± 1/2 festoon)	“Mostly” reaching posterior margin of scutum
III	USA (AZ), MX	Present	“Mostly” wider	1 spine + 1 fine seta	Comma-shaped, elongated, with narrow projection (± 1/2 festoon)	Comma-shaped, with narrow projection (± 1/3 festoon)	Reaching posterior margin of scutum
IV	PE, CH, EC	Present	Wider	2 spines of unequal length	Comma-shaped, elongated, with narrow projection (± 1/2 festoon)	Comma-shaped, with narrow projection (± 1/2 festoon)	“Mostly” reaching posterior margin of scutum

*Abbreviations*: AR, Argentina; BR, Brazil; CO, Colombia; CR, Costa Rica; VE, Venezuela; USA, United States; AZ, Arizona; MX, Mexico; PE, Perú; CH, Chile; EC, Ecuador

Analyses of the individual gene sequences, commonly used to reassess taxonomic issues within *Amblyomma* taxa [36, 39–41], were unable to resolve relationships within the group. Only analyses of the concatenated datasets could subdivide the *A. maculatum - A. triste* group into three supported clades characterized by distinct geographical ranges, but not always by distinct morphologies since Morphotype III is not monophyletic but is instead a basal polytomy within the Morphotype II+III cluster. The genetic similarity, often identity, particularly between mitochondrial sequences from eastern USA and Arizona-Mexico (Additional files 1, 2, 3, 4: Tables S1-S4) indicate that the spurs and the features of the spiracular plate commonly used to distinguish *A. maculatum* from *A. triste* are not species-specific characters. Molecular divergence values between morphotypes II and III are equivalent to diversity within Morphotype I, further indicating that the two morphologically distinct but genetically uniform North American populations, can be considered to belong to the same species, *A. maculatum*, which extends into parts of northern South America. Although we only obtained sequences from a single Colombian sample of *A. maculatum*, it consistently clustered with a sample from Georgia, USA (21D) as a slightly separated lineage within the Morphotype II+III clade, confirming the occurrence of the species in Colombia, as recently shown in a study on the diversity of Colombian ticks [21].

If the morphology of the tibial armature and of the spiracular plates cannot consistently be associated with speciation, the next question is whether we should consider Morphotype I, i.e. *A. triste* (*s.s.*), and Morphotype IV to be different species from *A. maculatum*. Genetic distance between populations of ticks that are geographically remote or even disjunct in their distribution is to be expected. Nevertheless, even the largest distances within the *A.maculatum - A. triste* group are comparable to intraspecific distances in other *Amblyomma* [36, 39, 40] and *Ixodes* [42–44] species when corresponding gene distances are compared separately. Nevertheless, mutation rates can vary between more or less related lineages and comparisons of simple distance values between distant taxa can be misleading. In this case, however, we have verified that within the *A. maculatum* group mutation rates are not significantly different between the sister lineages. Because distance values within Morphotype I (0.91–1.22%) are equal to or higher than distances between the same Morphotypes I and II+III (0.32–0.71%), we deduce that *A. triste* should also be returned to a junior synonym of *A. maculatum* (priority by page number in [4]). Although distances between Morphotype IV and the other morphotypes are slightly higher (1.22–1.70%), they are significantly lower than those between *A. tigrinum* and all morphotypes (3.91–4.31%).

In this study we are facing a situation in which morphological and molecular data are not offering a congruent solution, in a taxon (the ingroup) that radiated very recently. Our data do not support consistently the subdivision of this group into four species, although we agree that some of the presented evidence would provide justification. For now, we can only base our conclusion on our results, but there is no doubt that additional studies at a finer taxonomic level based either on nuclear codominant very variable markers (microsatellites or SNPs) and cross-breeding experiments will help in reaching more satisfying conclusions.

In the case of *A. tigrinum*, this species differs from *A. triste* and *A. maculatum* by host preferences [17, 45–47], ecological preferences [17] and morphology of both adults and immature stages [17]. Molecularly, *A. tigrinum* sequences always clustered in a well-defined monophyletic lineage. Although divergence values between *A. tigrinum* and *A. maculatum - A. triste* are moderately higher than intraspecific values, they remain much lower than the interspecific distances recorded between outgroup species, and between outgroup and ingroup taxa. More importantly, the variable nuclear gene used in this study (ITS2) which has successfully been used for taxonomic reassessments among South American *Amblyomma* species [36, 41], like some of the mitochondrial genes, included *A. tigrinum* in a polytomic ingroup and did not support a clear split between the taxa. For the time being, we do not consider *A. tigrinum* to be a synonym of *A. maculatum*, as proposed by Neumann [6] but to be a separate taxon of very recent radiation from *A. maculatum - A. triste*. After all, it has been shown that in some cases differences in host association can trigger rapid genetic divergence in tick species [48]. Only cross-breeding experiments are likely to determine whether the time elapsed since the divergence of the two lineages (2.1 Mya) has been sufficient for them to become different species.

In terms of node dating, the *A. maculatum - A. triste* radiation initiated no earlier than 700,000 years ago, a time frame not too different from that evaluated for the intraspecific diversification of *A. variegatum* [39] and of *Ixodes scapularis* Say [43, 44]. The branch lengths within the *A. maculatum - A. triste* clade are very short compared to those between the ingroup and outgroup and between different outgroup species (Fig. 8). This indicates that the radiation from the earliest common ancestor of the *A. maculatum* group was very rapid, almost explosive. If we compare our tree topology to that of other *Amblyomma* groups (the *A. cajennense* and *A. parvum* groups) with similar geographical distributions [36, 40], it appears that the *A. maculatum* group colonized its present range much later and very rapidly. Alternatively, we might consider the possibility that earlier widespread populations went almost extinct and that the present lineages arose through a major bottleneck. It could be argued that Morphotypes II-III-IV have evolved along the same biogeographical patterns described for *A. mixtum* [36] a species which exploited the closing of the Panama Isthmus (3 Mya) for dispersal through a wide area encompassing Colombia, the coast of Ecuador, Central America and southern North America. This could speculatively have been followed by allopatric incipient diversification occurring along the western coast of northern South America (Peru), in the Madrean Archipelago (Sky Islands) north of Mexico and in southern Arizona [49], while the most common morphotype (II) survived as such in Colombia and southeastern North America. Vicariant events cannot, however, explain the split between Morphotype II and I.

Additional studies with different molecular markers and many more samples, could help to better reconstruct the demographic history of the group.

## Conclusions

In summary, the data and evidence presented are inconsistent with the hypothesis that *A. maculatum*, *A. triste* and *A. tigrinum* represent three separate species. The evidence presented in this study supports the conspecificity of *A. maculatum* and *A. triste.* It is possible that the minor observed morphological differences are the result of a very rapid adaptation to slightly different environments not yet associated with sufficient genetic differentiation to support speciation. Further studies, especially cross-breeding experiments, should follow, as they may add valuable information and further support (or reject) the hypothesis of conspecificity of *A. maculatum* and *A. triste* raised in this study.

## Methods

### Morphological analysis

Morphological analyses were performed through examination of adult ticks deposited in the following tick collections: (i) Tick Collection of the Instituto Nacional de Tecnología Agropecuaria, Estación Experimental Agropecuaria Rafaela, Rafaela, Argentina (INTA); (ii) U.S. National Tick Collection, Georgia Southern University, Statesboro, Georgia, USA (USNTC, RML accession numbers); (iii) Tick Collection of the Departamento de Parasitología Veterinaria, Facultad de Veterinaria, Universidad de la República, Salto, Uruguay (DPVURU); and (iv) Coleçao Nacional de Carrapatos da Faculdade de Medicina Veterinaria e Zootecnia, Universidade de São Paulo, Brazil (CNC). Ticks determined as *A. triste*, *A. maculatum* and *Amblyomma* sp. cf. *A. triste - A. maculatum* from Argentina, Brazil, Chile, Colombia, Ecuador, Guatemala, Honduras, Mexico, Nicaragua, Peru, Uruguay, USA and Venezuela, were included in the morphological analysis. We also have examined the type-specimens of *A. triste*, *A. maculatum* and *A. tigrinum* from the Zoological Museum in Berlin (ZMB). The terminology used in the morphological diagnoses follows Nava et al. [17].

### Sampling for molecular analyses

Our sample included a total of 109 adult specimens initially identified as *A. maculatum*, *A. triste* or *A. tigrinum*, and two specimens identified as *A. parvitarsum* and *A. neumanni. Amblyomma maculatum* ticks were from the USA (Georgia and Florida), Peru and Colombia; *A. triste* were from Argentina, Brazil, Uruguay, Peru, Mexico and Arizona (USA); *A. tigrinum* were from Argentina and Brazil; and *A. parvitarsum* and *A. neumanni* were from Argentina. When available, specimens from several localities were included to consider variation between and within different regions (Table 1). The ticks were subsequently reclassified by using a newly outlined phenotypic subdivision (see Results section) of *A. maculatum - A. triste* into four morphotypes (Table 1). Ticks were obtained from 28 localities and 7 countries (Fig. 1), and coded as follows: Argentina (BA, Buenos Aires; CR, Corrientes; FO, Formosa; SDE, Santiago del Estero), Brazil (GO, Goiás; MGS, Mato Grosso do Sul; SP, Sao Paulo), Colombia (SR, Santander), Peru (PU) (IC, Ica; TU, Tumbes; PI, Piura), Mexico (MX) (HO, Sonora), and the USA (FL, Florida; GA, Georgia; AZ, Arizona) (Table 1).

### DNA extraction, PCR and sequencing

Tick DNA was extracted and, when possible, the exoskeletons were preserved for further morphological analysis following previously published protocols [39, 50]. A small portion of the postero-lateral idiosoma of each tick was removed by using a sterile disposable scalpel and the tick was incubated overnight in 180 μl Qiagen ATL lysis buffer (Qiagen, Valencia, CA, USA) and 40 μl of a 14.3 mg/ml solution of proteinase K (Roche Applied Sciences, Indianapolis, IN, USA). After repeated vortexing and ascertaining that the lysis was complete, each exoskeleton was stored in 70% ethanol and kept as a voucher specimen. The lysed tissues were further processed as previously described [36, 39, 50]. Five mitochondrial gene sequences, *12S* rDNA (small subunit ribosomal RNA), *16S* rDNA (small subunit ribosomal RNA), *cox*1 (cythochrome *c* oxidase subunit 1), the control region or d-loop (DL) were amplified with previously reported sets of primers [36, 39, 50–52]. In addition, a portion of the nuclear ribosomal internal transcribed spacer 2 (ITS2) was amplified by slightly modifying a previously published protocol to include 35 instead of 27 annealing cycles [39, 53]. PCRs were performed using a MasterTaq kit (5-Prime, Gaithersburg, MD, USA). Each reaction contained 2.5 μl of tick DNA, 2.5 μl of 10× Taq buffer, 5 μl of 5× TaqMaster PCR Enhancer, 1.5 μl of MgAc (25 mM), 0.5 μl of dNTP mix (10 mM each), 0.1 μl of Taq polymerase (5U/μl), 1.25 μl of each primer from a 10 pmol/μl stock solution (Invitrogen, Life Technologies Corporation, Grand Island, NY, USA) and 14.6 μl of molecular biology grade water. The two DNA strands of each amplicon were purified and sequenced at the High-Throughput Genomics Unit (HTGU, University of Washington, Seattle, WA, USA or at Eurofins, Louisville, KY, USA) and were assembled with Sequencer 4.5 (Gene Codes Corporation, Ann Arbor, MI, USA).

### Phylogenetic analyses

Sequences were manually aligned with McClade 4.07 OSX (Sinauer Associates, Sunderland, MA, USA) [54]. Secondary structure was considered in aligning ribosomal genes [50] and DL [55]. Codon organization was taken into account when aligning the *cox*1 data set. Each data set was analyzed by maximum parsimony (MP) with PAUP [56] and by Bayesian inference analysis (BI) using MrBayes 3.2.4 [57, 58]. Branch support was assessed by bootstrap analysis (1000 replicates) with PAUP for MP, and by posterior probability with MrBayes for BI. MP heuristic searches were performed by branch-swapping using the tree bisection-reconnection (TBR) algorithm. Maximum likelihood distances were calculated after the nucleotide substitution model best fitting the data was selected by JModeltest v.2.1.7 [59, 60]. Maximum likelihood pairwise distances were calculated based in the selected model by using PAUP. Two runs with four chains each were run simultaneously for BI analyses (1,000,000 generations). Trees were sampled every 100 iterations. Trees saved before the average standard deviation of split fragments converged to a value < 0.01 were discarded from the final sample. When necessary, the number of generations was increased so that the number of discarded samples would not exceed 25% of the total sampled trees. The 50% majority-rule consensus tree of the remaining trees was inferred, and posterior probabilities recorded for each branch. Two concatenated datasets, one (mtDNA) including 4 mitochondrial genes (*12S* rDNA, *16S* rDNA, DL and *cox*1) and one including the same 4 gene sequences and ITS2 sequences (mt+nDNA), were analyzed following the same procedure outlined for the separate analyses. *A. parvitarsum* and *A. neumanni* were used as outgroups in our analyses because they are recognized as being close relatives of our ingroup taxa [3]. Additional species were considered as possible outgroups and preliminary analyses were performed with the following taxa: *A. aureolatum*; *Amblyomma coelebs* Neumann, 1899; *Amblyomma dubitatum* Neumann, 1899; *Amblyomma oblongoguttatum* Koch, 1844; and *A. ovale.* These alternative outgroups were discarded because they were too distantly related to the ingroup to provide resolution within ingroup taxa.

### Molecular clock and divergence dates

Additional outgroup sequences available for the 6 species of *Amblyomma* species now included in the *A. cajennense* complex [*Amblyomma cajennense* (Fabricius, 1787), *Amblyomma sculptum* (Berlese, 1888), *Amblyomma mixtum* (Koch, 1844), *Amblyomma tonelliae* (Nava, Beati & Labruna, 2014), *Amblyomma patinoi* (Labruna, Nava & Beati, 2014), and *Amblyomma interandinum* (Beati, Nava & Cáceres, 2014)] were incorporated in a concatenated dataset (*12S* rDNA, DL and ITS2), including the unique *A. maculatum*, *A. triste* and *A. tigrinum* sequences. *Amblyomma cajennense* has been shown to be sufficiently close to the *A. maculatum* group to serve as alternate outgroup [3], with the additional advantage of having already been analyzed in terms of phylogeographical evolution and node dating [36]. In order to test substitution rate variation among lineages, relative-rate tests were applied to the main sister clades (*A. tigrinum vs A. maculatum - A. triste*, Peruvian *vs* all other clades, southern South American *vs* North American) by using DAMBE [61]. In addition, the molecular clock hypothesis was tested by the least square method and the likelihood ratio-test also in DAMBE [61]. A tentative estimate of the divergence time for the *A. maculatum* group of species was performed by using the relaxed molecular clock model implemented in BEAST v.1.7.4 [62, 63]. Monophyly was constrained for clades supported in the phylogenetic analysis. The tree prior was set to Calibrated Yule Process, and the molecular clock set to uncorrelated lognormal distribution. Chain lengths were set to 10,000,000 and data were sampled every 1000 iterations with a random starting tree. After deleting 5% of the generated trees, the remaining trees were summarized in a combined 50% maximum clade credibility tree by using TreeAnnotator v.1.7.4. Data were analyzed with Tracer v.1.6.0 and FigTree v.1.3.1 was used to visualize tree structure, with mean divergence times. Node calibration was based on previously generated estimates for the *A. cajennense* complex [36, 64], with the node at the origin of the *A. cajennense* radiation set at 17 ± 1.5 Mya.

## Additional files

Additional file 1: **Table S1.** Geographical distribution of the *12S* rDNA haplotypes. *Abbreviations*: PI, Piura; TU, Tumbes; GA, Georgia; FL, Florida; AZ, Arizona; SDE, Santiago del Estero; BA, Buenos Aires; CR, Corrientes; FO, Formosa; MGS, Mato Grosso do Sul; SP, São Paulo; GO, Goias; $, *A. maculatum*; %, *A. triste*; #, *A. triste - A. maculatum*; *, *A. tigrinum*. (XLSX 11 kb)

Additional file 2: **Table S2.** Geographical distribution of the *16S* rDNA haplotypes. *Abbreviations*: PI, Piura; TU, Tumbes; IC, Ica; GA, Georgia; FL, Florida; AZ, Arizona; SDE, Santiago del Estero; BA, Buenos Aires; CR, Corrientes; FO, Formosa; MGS, Mato Grosso do Sul; SP, São Paulo; GO, Goias; $, *A. maculatum*; %, *A. triste*; #, *A. triste - A. maculatum*; *, *A. tigrinum*. (XLSX 13 kb)

Additional file 3: **Table S3.** Geographical distribution of the *cox*1 haplotypes. *Abbreviations*: PI, Piura; TU, Tumbes; IC, Ica; GA, Georgia; FL, Florida; AZ, Arizona; SDE, Santiago del Estero; BA, Buenos Aires; CR, Corrientes; FO, Formosa; MGS, Mato Grosso do Sul; SP, São Paulo; GO, Goias; $, *A. maculatum*; %, *A. triste*; #, *A. triste - A. maculatum*; *, *A. tigrinum*. (XLSX 13 kb)

Additional file 4: **Table S4.** Geographical distribution of the DL haplotypes. *Abbreviations*: PI, Piura; TU, Tumbes; IC, Ica; GA, Georgia; FL, Florida; AZ, Arizona; SDE, Santiago del Estero; BA, Buenos Aires; CR, Corrientes; FO, Formosa; MGS, Mato Grosso do Sul; SP, São Paulo; GO, Goias; $, *A. maculatum*; %, *A. triste*; #, *A. triste - A. maculatum*; *, *A. tigrinum*. (XLSX 12 kb)

Additional file 5: **Table S5.** Geographical distribution of the ITS2 genotypes. *Abbreviations*: PI, Piura; TU, Tumbes; GA, Georgia; FL, Florida; AZ, Arizona; BA, Buenos Aires; CR, Corrientes; FO, Formosa; MGS, Mato Grosso do Sul; SP, São Paulo; GO, Goias; SDE, Santiago del Estero; $, *A*. *maculatum*; %, *A. triste*; #, *A.triste - A. maculatum*; *, *A. tigrinum*. (XLSX 10 kb)

## Abbreviations

BI: Bayesian inference
MP: Maximum parsimony
mtDNA: Mitochondrial DNA
Mya: Millions of years ago
nDNA: Nuclear DNA
